# Findings of multiple HPV genotypes in cervical carcinoma are associated with poor cancer-specific survival in a Swedish cohort of cervical cancer primarily treated with radiotherapy

**DOI:** 10.18632/oncotarget.24666

**Published:** 2018-04-10

**Authors:** Malin Kaliff, Bengt Sorbe, Louise Bohr Mordhorst, Gisela Helenius, Mats G. Karlsson, Gabriella Lillsunde-Larsson

**Affiliations:** ^1^ Department of Laboratory Medicine, Faculty of Medicine and Health, Örebro University, Örebro SE 701 82, Sweden; ^2^ Department of Oncology, Faculty of Medicine and Health, Örebro University, Örebro SE 701 82, Sweden

**Keywords:** cervical cancer, HPV, recurrences, survival

## Abstract

Cervical cancer (CC) is one of the most common cancers in women and virtually all cases of CC are a result of a persistent infection of human papillomavirus (HPV). For disease detected in early stages there is curing treatment but when diagnosed late with recurring disease and metastasis there are limited possibilities. Here we evaluate HPV impact on treatment resistance and metastatic disease progression.

Prevalence and distribution of HPV genotypes and HPV16 variants in a Swedish CC patient cohort (n=209) was evaluated, as well as HPV influence on patient prognosis. Tumor samples suitable for analysis (n=204) were genotyped using two different real-time PCR methods. HPV16 variant analysis was made using pyrosequencing.

Results showed that HPV prevalence in the total series was 93%. Of the HPV-positive samples, 13% contained multiple infections, typically with two high-risk HPV together. Primary cure rate for the complete series was 95%. Recurrence rate of the complete series was 28% and distant recurrences were most frequent (20%). Patients with tumors containing multiple HPV-strains and particularly HPV genotypes belonging to the alpha 7 and 9 species together had a significantly higher rate of distant tumor recurrences and worse cancer-specific survival rate.

## INTRODUCTION

Cervical cancer (CC) is internationally one of the most common cancers in women, in 2012 over 500 000 new cases were estimated and in several developing countries it is the most common cause of cancer-related mortality in women [1]. In Sweden the incidence of CC has been reduced by half since the 1970s much due to the national screening program. Today, the incidence of CC in Sweden is about 11/100 000 women [2, 3].

The majorities (∼80%) of CC are squamous cell carcinomas (SCC), which typically develop in the transition zone where endocervical glandular cells meet ectocervical squamous cells. The second most common histological subtype is adenocarcinomas (AC) (∼20%), occurring in the endocervix and therefore being harder to detect in an early stage. Other epithelial carcinomas, like adenosquamous carcinomas (ASC), account for a small percentage of the total number of CC. There has been an increase in incidence of AC in CC, both in total and in proportion to SCC [4, 5]. Virtually all cases of CC are a result of a persistent infection of human papillomavirus (HPV). In humans, over two hundred genotypes of HPV have been found [6], however only a fraction of these have been shown to be carcinogenic. HPV are double-stranded DNA viruses that are divided into five genera based on differences in the nucleotide sequence in the well conserved L1 gene [7]. The HPV genotypes infecting mucosa belong to the alpha-papilloma genera and International Agency for Research on Cancer (IARC) have identified thirteen: group 1 (HPV16, 18, 31, 33, 35, 39, 45, 51, 52, 56, 58, 59 and 66) as carcinogenic, group 2A (HPV68) as probably carcinogenic and group 2B (HPV26, 53, 67, 70, 73 & 82) as possible carcinogenic. HPV genotypes in the alpha-papilloma genera linked to cancer development are recognized as mucosal high risk genotypes (hrHPV), in contrast to the mucosal low risk genotypes (lrHPV) as for example HPV6 and 11 that can cause genital warts. The majority of the mucosal high risk genotypes involved in cancer development belong to alpha 7 (HPV18, 39, 45, 59) or 9 (HPV16, 31, 33, 35, 52, 58) species and a few genotypes belong to alpha species 5 (HPV51) and 6 (HPV56, 66) [8]. An infection with HPV16, 18, 31 or 33 constitutes the largest risk of developing cervical intraepithelial neoplasia 3 (CIN3), the precursor of CC [9] and HPV16 and 18 are also the genotypes most common in CC, found in over 70% of all cervical tumors. HPV genotype-variants share more than 98% of their DNA sequence. HPV16 have been divided into variants named after their geographical prevalence. The four major HPV16 variant lineages are European-Asian (EAS), African-1 (AFR-1), African-2 (AFR-2) and North-American/Asian-American (NA/AA). The European variant lineage is further arranged into several variant sublineages [10, 11]. Diverse HPV16 variants have in studies shown to contribute to the development of cervical cancer and precancerous lesions to different degrees [12–14].

Specific HPV genotypes causing CC have been linked to poor prognosis in the patient. For example, HPV18 have in a number of studies been associated with high level of disease recurrence and poor survival [15, 16], while other studies failed to distinguish between the genotypes in regards to prognosis [17, 18]. There are also reports of poor prognosis for patients with cervical carcinomas containing multiple HPV strains, but data are limited [16, 19].

### Aim

The aim of this study was to evaluate the prevalence and distribution of HPV genotypes and HPV16 variants in a Swedish patient cohort, diagnosed with cervical carcinoma and treated with radiation therapy. The study also aimed to assess HPV genotype and HPV16 variant influence on the patient prognosis.

## RESULTS

### Patient characteristics and HPV genotyping

**Figure 1:**
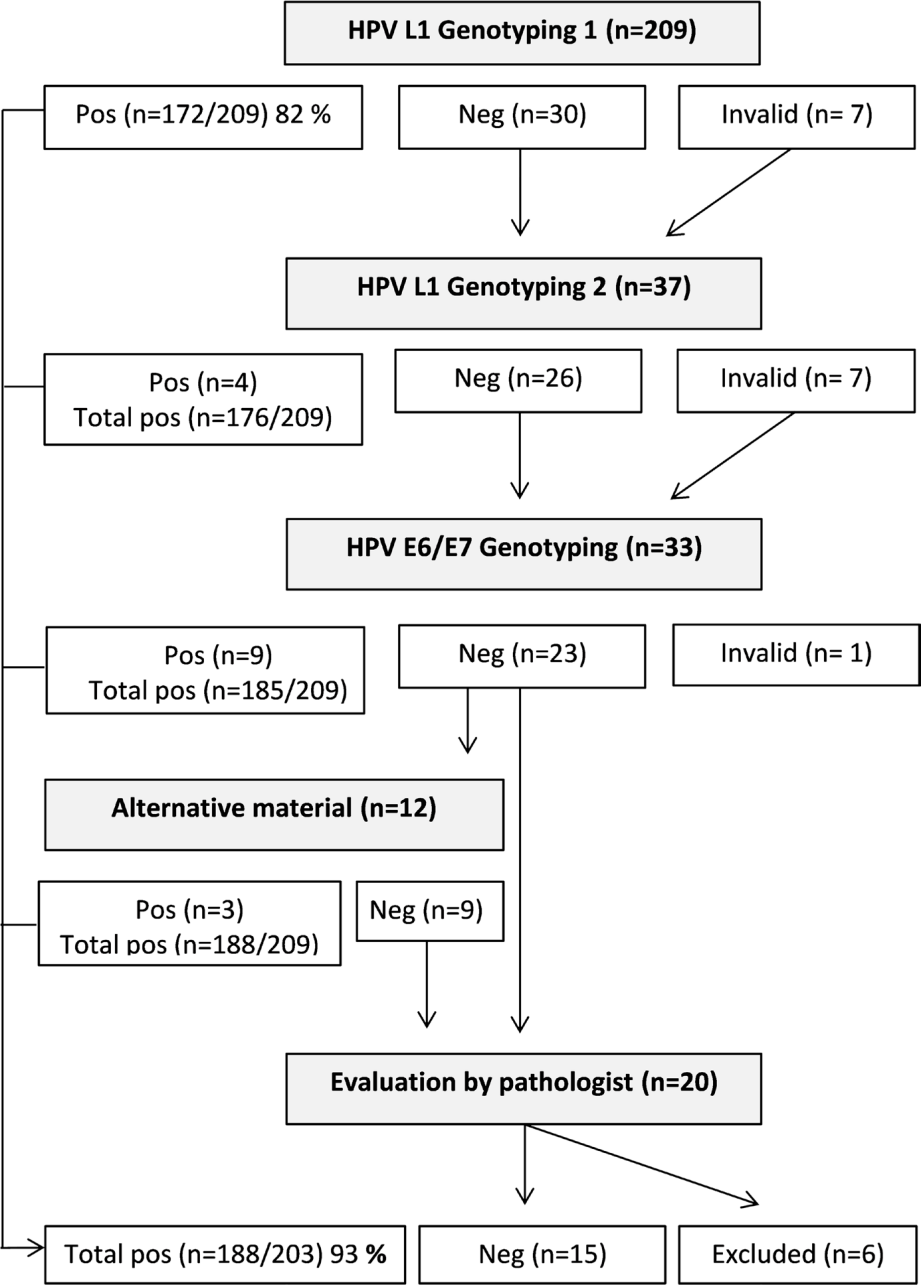
Study design flow-chart Tumors from the total study population (n=209) were tested with HPV L1 Genotyping method. All samples with negative or invalid results were rerun (n=37). Samples with two-time negative or invalid results using the L1 based approach were analyzed with a HPV E6/E7 genotyping method (n=33). Patient samples with negative results after both genotyping methods and with available alternative tissue block were reanalyzed twice with method targeting L1 and once with methods targeting E6/E7 (n=12). HPV negative patient samples without alternative material available or with a consistent negative result after genotyping were evaluated by pathologist (n=20). After repeated testing, use of alternate genotyping approach and exclusion of reclassified cases or cases with insufficient tumor material, 93% (188/203) of the tumors were positive for HPV.

Five patients of the total study population (n=209) were excluded due to inadequate tumor tissue samples or histological reevaluation. In the total series of 204 patients the mean age was 60 years (range 23-90). Most tumors were SCC (84%, n=172), followed by AC (14%, n=28) and ASC (2%, n=4). By FIGO staging, 44 (22%) tumors were stage I, 125 (61%) tumors stage II, and 24 (12%) tumors stage III and 11 (5%) tumors stage IV. Genotyping results could be obtained in 203 out of the 204 samples and the sample with invalid genotyping result was excluded. After repeated testing, use of alternate genotyping approach and exclusion of reclassified cases, 93% (188/203) of the tumors were positive for HPV (Figure 1). The most frequently detected genotype was HPV16 (43%) followed by HPV18 (16%), HPV31 (8%), HPV45 (8%) and HPV33 (6%) (Table 1). Most HPV45 positive tumors were SCC (15/17) 88% while HPV18 was significantly (Pearson chi-square; P < 0.0001) more common in AC (50%) than in SCC (14%). Single infections was detected in 163 tumors (87%) and 25 tumors (13%) carried multiple infections. The majority of the tumors with multiple infections tested positive for two or three hrHPV except eight tumors that contained hrHPV together with intermediate risk- or lrHPV (Table 1). Tumors with multiple HPV infections in this cohort were evenly distributed over time. In the 1990s 12% (7/60) of the tumors harbored multiple infections, in the 2000s 14% (11/80) and in the 2010s 11% (7/64).

**Table 1:** Total prevalence and distribution of HPV genotypes

HPV-type	Genotyping results	HPV-type	Sample results	HPV-type	Sample results
*Single infections hr/imr/lr*	Single/Total	*Multiple infections hr*		*Multiple infections hr+imr/lr*	
**IARC 1**		**IARC 1**		**IARC 1+ IARC 2A/2B/lr**	
**16**	78/93	16+18	2	16+40	1
**18**	30/35	16+31	4	16+42	1
**31**	8/17	16+33	1	18+44	1
**33**	8/12	16+35	1	16+68	1
**35**	1/2	16+39	1	31+45+68+53+43+54	1
**39**	4/5	16+45	1	33+42	1
**45**	12/17	16+56	1	56+53	1
**51**	2/3	16+31+45	1	58+42	1
**52**	3/3	18+31	1	**Total**	**8**
**56**	2/4	18+33	1		
**58**	3/4	31+45	2		
**59**	5/5	33+51	1		
**IARC 2A+2B**		**Total**	**17**		
**53**	0/2				
**70**	2/2				
**73**	2/2				
**68**	3/5				
**lr**					
**40**	0/1				
**42**	0/3				
**43**	0/1				
**44**	0/1				
**54**	0/1				
**Total**	**163/218**				

Of the 188 HPV-positive samples, single infections were present in 163 samples and multiple infections in 25 (17+8). Classification according to IARC (International Agency for Research on Cancer), hr=high risk, imr=intermediate risk, lr=low risk.

Of the tumors positive for HPV, 28% (52/188) harbored hrHPV genotypes belonging to the alpha 7 species, 61% (114/188) harbored hrHPV belonging to alpha 9 species and 5% (10/188) of the tumors harbored hrHPV of both alpha species. In the alpha 7 positive group, multiple infections were present in 2% (1/52) of the tumors, while in the alpha 9 positive group multiple infections were present in 11% (13/114) of the cases. Only a few of the tumors positive for hrHPV harbored HPV belonging to the alpha groups 5 and 6.

Patient characteristics (mean age, type of histology (SCC vs. AC/ASC), tumor size (mm) and tumor stage (early: FIGO I-II vs. advanced: FIGO III-IV) did not differ between patients with tumors containing single HPV genotypes compared to multiple genotypes or patients with tumors containing HPV from one of the alpha 7 or 9 species compared to both alpha 7 and 9 species (Table 2).

**Table 2:** Patient and tumor characteristics in: HPV single vs. multiple, HPV alpha 7 or 9 vs. alpha7+9 containing tumors

	Single infections	Multiple infections	Statistics
**Mean age (years)**	58.9	63.4	t-test; p=0.204
**Mean tumor size (mm)**	41.6	43.3	t-test; p=0.594
**Type of histology**			
SCC	144/163 (88%)	22/25 (88%)	Chi-square test; p=0.960
AC	19/163 (12%)	3/25 (12%)	
***Total***	**163**	**25**	
**Tumor Stage**			
Early stage (FIGO I-II)	138/163 (85%)	19/25 (76%)	Chi-square test; p=0.277
Advanced stage (FIGO III-IV)	25/163 (15%)	6/25 (24%)	
***Total***	**163**	**25**	

	HPV Alpha 7/9	HPV Alpha 7+9	Statistics
**Mean age (years)**	59.0	64.3	t-test; p=0.333
**Mean tumor size (mm)**	41.8	46.8	t-test; p=0.267
**Type of histology**			
SCC	147/166 (89%)	7/10 (70%)	Chi-square test; p=0.085
AC	19/166 (11%)	3/10 (30%)	
***Total***	**166**	**10**	
**Tumor Stage**			
Early stage (FIGO I-II)	137/166 (83%)	8/10 (80%)	Chi-square test; p=0.838
Advanced stage (FIGO III-IV)	29/166 (18%)	2/10 (20%)	
***Total***	**166**	**10**	

The cervical HPV-positive single strain (n=163) and HPV-positive multiple strain (n=25) groups and the HPV alpha 7 or 9 (n=163) and alpha 7+9 (n=10) groups did not show any statistical significant differences in patient mean age at diagnosis, type of histology, tumor size or tumor stage. SCC- Squamous cell carcinoma, AC-Adenocarcinoma.

### Clinical outcome

The primary cure rate for the complete series was 95%. There was no significant difference in primary cure rate in regards to single or multiple infections (Pearson chi-square test; p = 0.258), comparisons between specific genotypes or alpha species groups (alpha 7 & 9 vs. alpha 7 or alpha 9, Pearson chi-square test; p = 0.374). Specifically, for HPV18 and HPV45, the primary cure rate was 100%.

Recurrence rate of the complete series was 28% and distant recurrences were most frequent (20%). The overall recurrence-rate was significantly higher for patients with tumors containing multiple HPV strains (44%) compared to tumors with single infections (24%) (Pearson chi-square; p=0.027). Also, overall recurrence-rate was significantly higher for patients with tumors containing HPV from both alpha species 7 and alpha 9 (80%) compared to alpha species 7 or 9 alone (25%) (Pearson chi-square test; p = 0.0002). The overall recurrence-rate for tumors positive for HPV18 and HPV45 was 46% and 47%, respectively.

There was a significant association (Pearson chi-square test; p = 0.0022) between multiple HPV infections and distant recurrences. The distant recurrence rate for single strain tumors was 15% while 40% for multiple-strain tumors. In a logistic multivariate regression analysis, presence of multiple HPV genotypes was still an independent and significant predictive factor for distant recurrences (OR = 4.003 [95% CI: 1.536-10.433]; p = 0.005) after correction for tumor stage, tumor size, and histology (Table 3). Distant recurrences were also significantly associated (Pearson chi-square; p < 0.00001) with tumors containing HPV from both alpha7 and 9 species together (80%) compared to HPV from one of the species: alpha 7 or 9 (16%). For local and loco-regional recurrences no significant associations were found.

**Table 3:** Multivariate analysis

Multivariate analysis - Logistic regression analyses			
Factor	Odds ratio	95% CI	p value
FIGO-stage (III-IV vs. I-II)	2.070	0.806-5.318	0.131
Tumor size (per mm)	1.015	0.983-1.048	0.354
Histology	3.181	1.142-8.857	0.027
HPV (multiple vs. single)	4.003	1.536-10.433	0.005

Multivariate analysis - Cox proportional hazard regression analyses			
Factor	Hazard ratio	95% CI	p value
FIGO-stage ( III-IV vs. I-II )	4.946	1.377-17.766	0.024
Tumor size (per mm)	1.019	0.990-1.049	0.202
Histology	3.300	1.592-6.841	0.001
HPV (multiple vs. single)	2.383	1.125-5.047	0.023

Logistic regression analyses of predictive factors for distant tumor recurrences and Cox proportional hazard regression analyses of prognostic factors for cancer-specific survival rate. Histology: Adenocarcinoma vs. squamous cell carcinoma.

**Figure 2:**
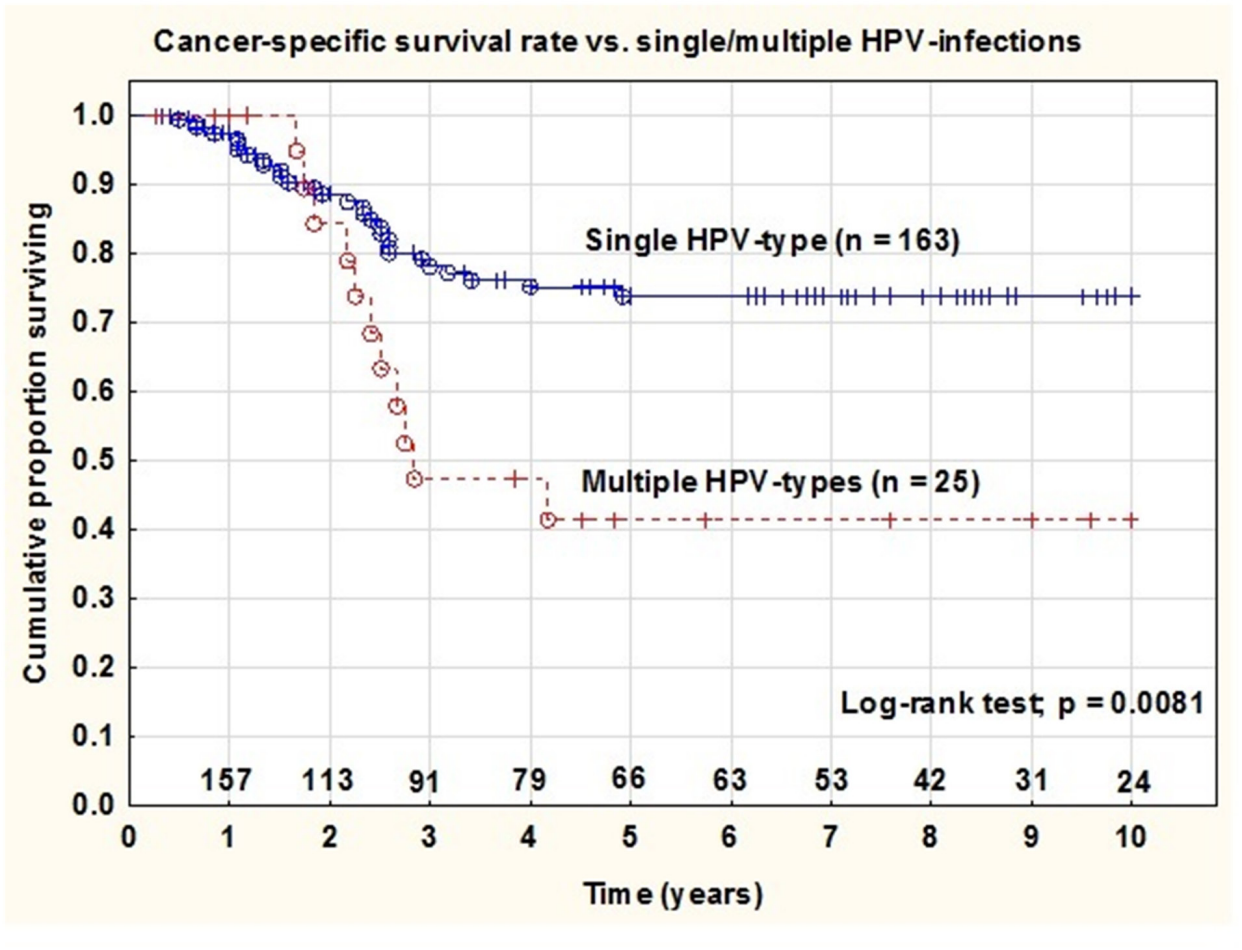
Cancer-specific survival rate in patients with tumors containing single vs. multiple HPV infections In patients diagnosed with cervical cancer and treated with radiation (at year 0 n=188) there was a statistical significant difference (p=0.0081) in survival rates between the two groups of patients with single strain HPV-positive tumors vs. multiple HPV-positive tumors.

The cancer-specific survival rate at 5 years for the complete series was 66% and was significantly (log-rank test; p = 0.0081) worse for patients with tumors containing multiple HPV genotypes (41%) compared to single HPV genotypes (74%) (Figure 2). In a Cox proportional hazard regression analysis tumor stage (stage IV) (HR=4.946 [95% Cl: 1.377-17.766]; p= 0.024), histology (AC) (HR=3.300 [95% Cl: 1.592-6.841]; p= 0.001) and infection with multiple HPV strains (HR=2.383 [95% Cl: 1.125-5.047]; p= 0.023) were independent and statistically significant prognostic factors (Table 3).

**Figure 3:**
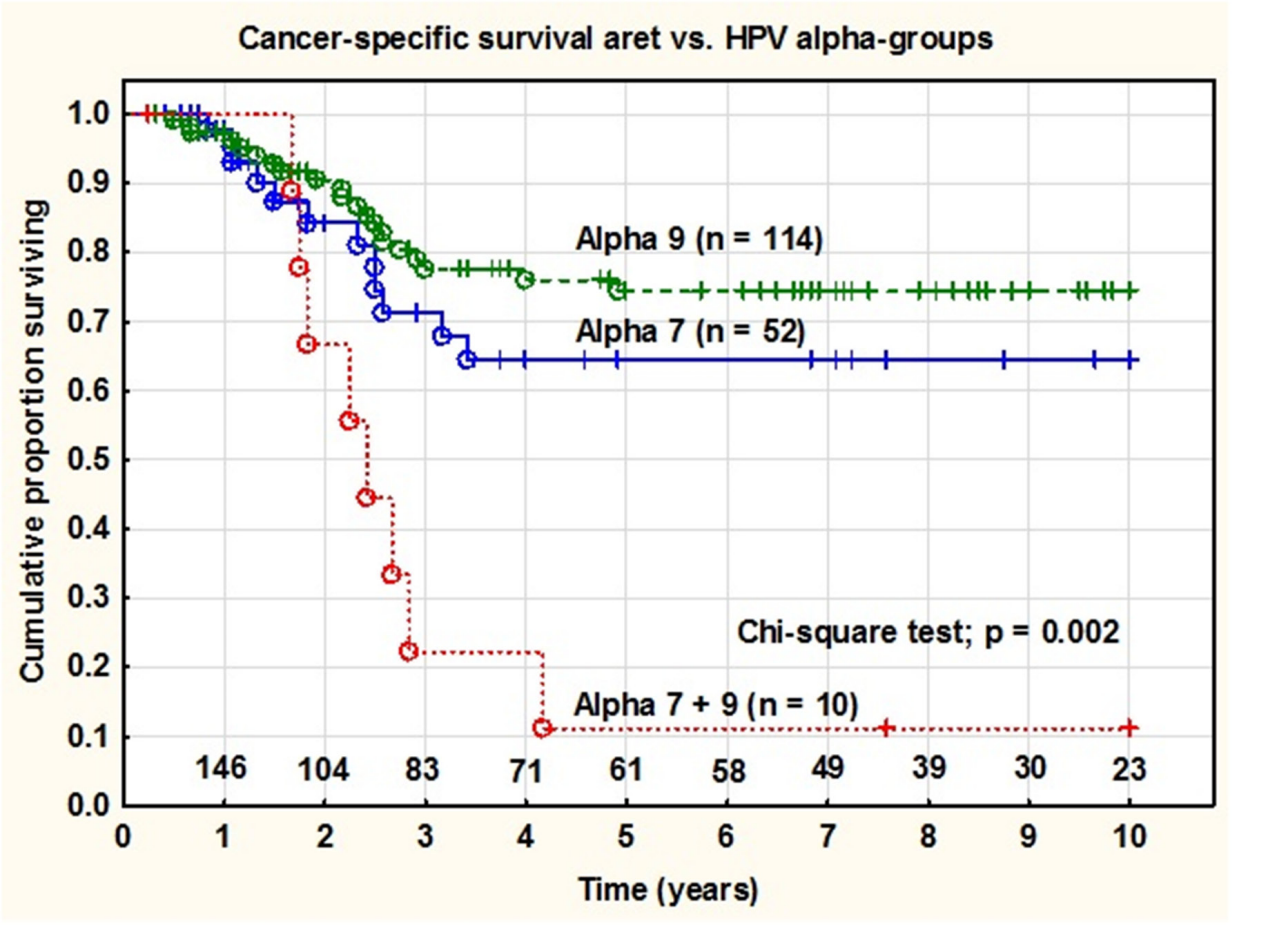
Cancer specific survival rate in patients with tumors containing HPV from alpha7, alpha9 and alpha7+9 infections There was a statistical significant difference (p=0.002) in survival rates between the groups of patients diagnosed with cervical cancer and treated with radiation (at year 0 n=176) with tumors containing HPV from alpha7, alpha9 and both groups together.

There were no significant differences in 5-year cancer-specific survival rate between tumors containing HPV types belonging to alpha 9 species (75%) and tumors containing alpha 7 species (65%) (log-rank test; p = 0.270). However, tumors containing HPV types belonging to both alpha 9 and alpha 7 (n = 10) had significantly worse 5-year cancer-specific survival (11%) (Pearson chi-square test; p = 0.002) compared to alpha 7 or 9 alone (Figure 3).

HPV18 positive tumors were associated with an inferior 5-year survival rate (49%) compared to HPV18 negative tumors (73%) (Log-rank; p = 0.016). HPV18 was significantly (Pearson chi-square; P < 0.0001) more common in adenocarcinomas (50%) than in squamous cell carcinomas (14%). There was no significant association between prognosis and any other individual HPV-genotype.

### HPV16-variant determination

Variant determination was successfully performed in 87/93 (94%) of HPV16 positive samples. The majority were of EAS origin: 94% (82/87), while 5% (4/87) belonged to the NA/AA lineage and 1% (1/87) to AFR1 lineage. Among the EAS lineage samples, all were of the European sublineage (EUR). Within EUR the HPV16 reference, European prototype (Ep) was most commonly found (n=36) followed by samples with E-G350 (n=34), EG131G (n=5), EG131 (n=3), and E-C109G (n=3) polymorphisms. One tumor contained a double infection of both E-G350 and Ep.

Despite few cases, EUR samples with E-G131G polymorphism had the lowest long-term survival probability, but with no significant differences from the other tested EUR samples (chi-square test; p = 0.239).

## DISCUSSION

This study aimed to evaluate the prevalence and distribution of HPV genotypes in a Swedish CC patient cohort, and also to assess HPV genotype and HPV16 variant influence on the patient prognosis. Detection and genotyping was initially made with a real time PCR method targeting the HPV L1 gene and samples with negative result were retested targeting the viral E6/E7 genes. Alternate genotyping approach was used to avoid false negative results due to disruption or loss of the initially targeted L1 gene, which could be a result of viral integration into the human genome [20]. The total prevalence of HPV in this cohort treated with radio therapy was 93%. The International Agency for Research on Cancer (IARC) have reported findings from three large systematic reviews involving 187 studies including over 20 000 cervical cancer patients showing a worldwide prevalence of HPV in cervical cancer to be 88% in SCC and 77% in AC [21]. In the present study, the most frequently detected genotypes were HPV16, 18, 31, 45 and 33. HPV16 was detected in 43% and HPV18 in 16% of the tumors. In comparison to IARC data [8], HPV16 prevalence was lower (43% vs 54%) while HPV33 and 45 prevalence were comparatively higher. Also, the proportion of HPV31, 33 and 45 in our study is somewhat higher comparing with findings in other European studies [22, 23].

Multiple infections were seen in 13% of all HPV positive cases, considerably higher than reported for a European multinational study by de *Sanjose et al* [23] (7%). In the study by de Sanjose, it is also described that the prevalence of multiple infections in invasive CC world-wide varies between 4% in North America to 19% in African groups. *Bachtiary et al* [19] on the other hand, found in their study from 2002 made in Austria the prevalence of multiple HPV in cervical cancer to be 44%. Comparison of proportion of multiple HPV genotypes in cervical cancer can be difficult since the multiple infection incidences depends on the number of HPV genotypes targeted. New and improved methods allow detection of an extended number of genotypes which gives an opportunity to investigate the impact of concurrent HPV genotypes, both high-, intermediate- and low risk HPV in cancer development and progression.

An ongoing HPV infection can be a risk factor for acquiring additional HPV infections [24, 25] and infection with multiple HPV genotypes is in turn a risk factor for persistent HPV infection [26, 27]; a prerequisite for developing CC. Interestingly, *Schmitt et al* [28] showed 2013 that multiple hrHPV infections were associated with cervical precancerous lesions in higher grade than single hrHPV infections.

In the present study, patients with tumors containing more than one HPV genotype had an unfavorable prognosis. Both significantly higher distant recurrent rate and worse cancer-specific survival rate was noted compared to patients with tumors containing single HPV genotypes. *Munugala et al* [16] reported cervical cancers with multiple infections containing at least one hrHPV to be associated with an almost five fold higher risk of radiation treatment failure compared to tumors containing single hrHPV infections. Similar findings were seen in a study by *Bachtiary et al* [19], but contradictory results are also present [29]. HPV positivity has been associated with better patient prognosis compared to HPV negativity in cancers of the head and neck [30], vagina [31] and cervical cancer [18]. In the latter example, HPV-negative tumors had a significantly higher occurrence of mutated p53 as well as lower survival rate. The viral E6 protein is known to degrade p53 which is thought to be an essential part of the apoptotic response to DNA damage after radiation. HPV-positive tumors response to radiation could be a result of a residue of p53 still active in spite of E6 degradation unlike in HPV-negative tumors where little to no wild type p53 remains [18, 30, 32]. Hypothesizing, multiple HPV infections in cervical tumors would result in increased levels of E6 and further decreased p53; this could impair the apoptotic response to radiation compared with single HPV tumors. Further analysis of p53 and viral load in tumors with multiple and single HPV infections would be of interest in our cohort.

Further, the results from this study show that patients with tumors harboring HPV genotypes from both alpha 7 and 9 species had a significantly higher distant recurrence rate and significantly lower 5-year cancer-specific survival rate compared to patients with tumors containing HPV genotypes from one of the alpha 7 or 9 species. The impact of HPV alpha species on prognosis has been sparsely investigated, but Wang et al [33] could in 2010 report that patients with tumors containing multiple HPV genotypes from alpha 7 species had the lowest survival rate, genotypes including alpha 7 and 9 together somewhat better and genotypes including alpha 9 most favorable outcome. In the present study, comparisons in prognosis between alpha groups could only be performed combining single and multiple HPV-positive tumors due to cohort number limitations. Despite, our findings provide a strong association between poor prognosis and infection with both alpha 7 and 9 species together, with a 5-year cancer specific survival rate at only 11%.

Despite the fact that most cervical carcinomas are the result of a persistent HPV infection, specific genotypes have shown to be more prone of risk for disease and progression. Here, HPV18-positivity was associated with inferior survival compared to tumors not containing HPV18. Similar results have been presented by Kim et al [34] from their cohort of women treated with radiation and also by Burger [15] and Munagala et al [16] who also show findings of worse survival and treatment failure for women with HPV18 positive tumors. Contradictory results have been presented by Cuschieri et al [17] where absence of HPV16 and/or HPV18 was associated with worse survival. HPV18 is consistently more associated with AC than SCC of the cervix [8, 23, 35] and cervical AC have in turn in several studies [36–38] shown to be associated with worse patient outcome compared to SCC. The impact of tumor histology is once again confirmed by our findings where the genotype HPV18 was significantly more common in AC tumors.

No significant impact of HPV16 variant influence on the patient prognosis was noted and nearly exclusively samples of EUR sublineages were found. A numeric low long-term survival was found in the E-G131G and NA/AA positive tumors samples. A similar finding of low survival association to the HPV16 E-G131 polymorphism has been shown in vulvar carcinomas [39], but the limited number of cases in both cohorts aggravates these findings and argues for larger study cohorts or combined datasets.

The cervical cancer incidence has been decreasing in the western countries and with primary and secondary prevention including vaccine and HPV screening it is believed to be a downward trend. In early stages of cervical cancer there is curing treatment with good prognosis however cervical cancer diagnosed in late stages and recurring disease with metastasis have poor prognosis and limited possibilities for effective treatment [40]. Evaluation of HPV impact on treatment resistance and metastatic disease progression is therefore needed in the upcoming challenge in developing efficient treatment alternatives. Also, and in relation to HPV vaccination, the impact of specific genotypes or groups of species needs to be further evaluated to improve patient prognosis.

We acknowledge some limitations with the present study. The study cohort includes patients diagnosed over two decades, where treatment standards have evolved which could potentially affect the outcome. A larger study group would potentially support accounted results.

In conclusion, we found in this Swedish series of cervical cancer treated with radiotherapy the HPV prevalence to be 93%. Of the HPV-positive samples, 13% contained multiple infections typically with two hrHPV together. Patients with tumors containing multiple HPV-strains and particularly HPV genotypes belonging to the alpha 7 and 9 species together had a significantly higher rate of distant tumor recurrences and a worse cancer-specific survival rate. Further analysis of p53 and HPV-viral load in cervical tumors containing multiple HPV could be of interest for investigating potential biological mechanisms.

## MATERIALS AND METHODS

The study population included 209 patients diagnosed with cervical cancer and treated with radiotherapy (external beam radiation and brachytherapy) between 1992 and 2014 at the Department of Oncology at Örebro University Hospital, Sweden. Of the total study population 204 patient samples were suitable for analysis. In this patient group 58% were treated with concomitant chemotherapy. The mean follow-up time of patients alive (n=113) was 61 moths (range 3-229 months) and the median follow-up times was 45 months. Patient samples were biopsies or resections collected from Örebro University Hospital, Uppsala University Hospital and the central hospitals in Eskilstuna, Falun, Gävle and Karlstad. Staging of the tumors were made at the time of diagnosis using the staging system of the International Federation of Gynecologists and Obstetricians (FIGO, Montreal 1994). Relevant clinical data were obtained from patient records at the Department of Oncology, Örebro University Hospital.

### Ethical approval

The study was given ethical approval by the regional ethics committee board in Uppsala (D nr 2008/122).

### DNA extraction

Formalin-fixed and paraffin-embedded (FFPE) tumor tissue samples were selected by a pathologist (MK) using slides made at time of diagnosis. Sections of tissue blocks (2-5, 10 μm cuttings) were de-waxed with xylene and used for DNA extraction with QIAamp DNA mini kit (Qiagen GmbH, Germany) following the manufacturer’s instructions.

### HPV L1 genotyping

Detection and genotyping was performed with Anyplex™ II HPV28 (Seegene, Seoul, Korea), a real time PCR method detecting 28 genotypes (HPV6, 11, 16, 18, 26, 31, 33, 35, 39, 40, 42, 43, 44, 45, 51, 52, 53, 54, 56, 58, 59, 61, 66, 68, 69, 70, 73 and 82) and the human gene Beta-globin (HBB). Amplicon lengths were between 100-200 bp within the viral L1 gene. The samples were run according to manufacturer's instruction as described before [41]. Anyplex™ II HPV28 has by EU-directive been approved to be used in *in-vitro* diagnosis with analysis of cervical swab and liquid based cytology specimens in EU. The method has previously been tested on archival FFPE material by Lillsunde et al and proven to be highly sensitive for FFPE samples [41]. All samples with negative or invalid results were rerun one time using DNA from the same extraction.

### HPV E6/E7 genotyping

Samples with two-time negative or invalid results using the L1 based approach (Anyplex™ II HPV28) were analyzed with a real-time PCR method targeting the viral genes E6 or E7. Reactions were carried out with a Taqman real-time PCR in eight separate reactions for every sample in the 7500 fast real-time PCR system (Applied Biosystems, Netherlands). Samples were analyzed for 12 hrHPV (HPV16, 18, 31, 33, 35, 39, 45, 51, 52, 56, 58, and 59), 2 lrHPV (HPV6 and 11) and the housekeeping gene β-globulin as previously described [31]. Results were analyzed in software 7500 fast system SDS (Applied Biosystems, Netherlands) and the curves were manually assessed using a threshold at 35 cycles for a positive result. Positive controls for HPV16 and human DNA was included as well as a non-template control.

Patient samples with negative results after both genotyping methods and with available alternative tissue block were reanalyzed twice with method targeting L1 and once with methods targeting E6/E7 according to procedure described above. HPV negative patient samples without alternative material available or with a consistent negative result after genotyping were evaluated by a pathologist (MK).

### Variant determination of HPV16

Samples positive for HPV16 were analyzed with PCR and pyrosequencing to determine HPV16 variants. Analysis was performed by targeting seven positions in the viral E6 gene (nt 109, 131, 132, 143, 145, 178 and 350; reference sequence NC_001526) [10]. Analysis was performed with PCR and pyrosequencing as previously described [39].

### Statistical analyses

Survival analyses were made using Kaplan-Meier survival methods and the curves were compared with the log-rank or Chi-square analysis. Comparisons of proportions were made with Person’s chi-square test and means were compared with independent t-test. Multivariate analysis of different prognostic factors was performed using the Cox proportional hazards model for survival outcome and logistic regression analyses for binary outcome. For the statistical tests a p < 0. 05 were considered to be statistically significant. For statistical analyses the Statistica software package (version 13, 2015; StatSoft, Inc, Tulsa, OK) was used.
